# The Microbiome of Meibomian Gland Secretions from Patients with Internal Hordeolum Treated with Hypochlorous Acid Eyelid Wipes

**DOI:** 10.1155/2022/7550090

**Published:** 2022-02-24

**Authors:** Shu Yang, Bing-Cheng Wu, Zhe Cheng, Lan Li, Yuan-Ping Zhang, Hui Zhao, Han-Mei Zeng, Dong-Fang Qi, Zi-Yao Ma, Jian-Guo Li, Rui Han, Fang-Zhou Qu, Yan Luo, Yi Liu, Xiao-Lei Chen, Hong-Mei Dai

**Affiliations:** ^1^Department of Ophthalmology, The First Hospital of Kunming, Kunming, 650011 Yunnan Province, China; ^2^Eye institute of Xiamen University, Xiamen, Fujian, China; ^3^Department of Ophthalmology, The Calmette Affiliated Hospital of Kunming Medical University, Kunming, China; ^4^Department of Ophthalmology, The Second Affiliated Hospital of Kunming Medical University, Kunming, China; ^5^Department of Ophthalmology, Shanghai General Hospital (Shanghai First People's Hospital), Shanghai 200080, China; ^6^Department of Pediatrics, The First Hospital of Kunming, Kunming, 650011 Yunnan Province, China; ^7^Department of Otorhinolaryngology, The First Hospital of Kunming, Kunming, 650011 Yunnan Province, China; ^8^Department of Endocrinology, The First Hospital of Kunming, Kunming, 650011 Yunnan Province, China

## Abstract

**Objective:**

The aims of our experiment were to compare the microorganisms in meibomian gland secretions from patients with internal hordeolum before and after treatment using hypochlorous acid eyelid wipes, to elucidate the mechanism underlying hypochlorous acid eyelid wipe treatment of internal hordeolum.

**Methods:**

This was a prospective, matched-pair study. A total of eight patients with internal hordeolum who attended the ophthalmology clinic of our hospital from April to August 2020 were included. Meibomian gland secretions were collected from subjects before treatment (Group A) and from patients cured after eyelid cleaning with hypochlorous acid eyelid wipes for 7 days (Group B). Samples were submitted to 16S rRNA high-throughput sequencing, and the resulting data were analyzed to compare the differences in the structure and composition of meibomian gland secretion microbial flora before and after treatment of internal hordeolum.

**Results:**

A total of 2127 operational taxonomic units were obtained from the two groups of samples, and there was no significant difference in alpha diversity before and after eyelid cleaning. At the phylum level, there was no significant difference between the two groups. The predominant phyla in Group A included the following: *Firmicutes* (32.78% ± 20.16%), *Proteobacteria* (26.73% ± 7.49%), *Acidobacteria* (10.58% ± 11.45%), *Bacteroidetes* (9.05% ± 6.63%), *Actinobacteria* (8.48% ±1.77%), and *Chloroflexi* (3.15% ± 3.12%), while those in Group B were the following: *Proteobacteria* (31.86% ± 9.69%), *Firmicutes* (29.07% ± 24.20%), *Acidobacteria* (11.33% ± 7.53%), *Actinobacteria* (7.10% ± 1.98%), *Bacteroidetes* (5.39% ± 5.17%), and *Chloroflexi* (3.89% ± 3.67%). Starting from the class level, significant differences in microbial communities were detected before and after eyelid cleaning (*P* < 0.05). Linear discriminant analysis effect size analysis showed the core flora in Group A microbiome comprising *Actinobacteria*, *Staphylococcus*, *Staphylococcaceae*, *Staphylococcus aureus*, *Ruminococcacea UCg-014*, *Ruminococcacea-UCG-014*, *Halomonadaceae*, *Neisseria, Methylobacterium, Frankiales, and Neisseria sicca*, *while those in Group B microbial* were *Streptococcus* sp., *Blautia*, *Bifidobacterium pseudocatenulatum*, *Subdoligranulum*, *Subdoligranulum variabile*, *Faecalibacterium*, and *Faecalibacterium prausnitzii*.

**Conclusion:**

Eyelid cleaning with hypochlorous acid eyelid wipes does not change the biodiversity in the meibomian gland secretions of patients with internal hordeolum. Hypochlorous acid eyelid wipes may affect the internal hordeolum through broad-spectrum antibacterial action to effectively reduce the relative abundance of symbiotic pathogens, such as *Staphylococcus*, *Neisseria*, *Actinomycetes*, and *Ruminococcus* and increase that of *Faecalibacterium prausnitzi*i and other symbiotic probiotics with anti-inflammatory effects.

## 1. Introduction

A hordeolum is an acute suppurative reaction within the eyelid glands that is usually staphylococcal in origin. According to different disease sites, hordeolum can be medically divided into external (Zeis gland or Moll gland) and internal (meibomian gland) types [1, 2]. This study focuses solely on the internal hordeolum. The clinical manifestations of internal hordeolum are a swollen red eyelid with a painful lump that appears spontaneously within a few days and purulent inflammation of the meibomian glands [3–5]. Lederman et al. reported that internal hordeolum pathogenesis was caused by the infection of the glands with *Staphylococcus* (*Staphylococcus epidermidis* or *Staphylococcus aureus*) [3, 4]. *Staphylococcus* is an opportunistic pathogen in the internal hordeolum, therefore reducing pathogen invasion is the key to treatment.

Nonsurgical treatments for internal hordeolum include hot compresses and topical or systemic antibiotics. Commonly used antibiotics include levofloxacin eye drops [5], ofloxacin eye ointment, and tobramycin eye drops plus dexamethasone eye ointment, among others [4, 6]. Although these antibiotics are effective for treatment of the disease, they have many limitations, including bacterial resistance [7], insufficient drug concentration of eye drops in the meibomian glands, ointment affecting patient visual perception, and side effects caused by long-term use of tobramycin dexamethasone eye ointment, such as increased intraocular pressure [8]. To address these problems, ophthalmologists have focused on hypochlorous acid, which has good bactericidal, anti-inflammatory, and wound healing effects [9–12], and is used as an antimicrobial agent for disinfection and treatment of diabetic feet [13], bedsores [14], and sinusitis [15], as well as skin disinfection [16, 17] and oral irrigation [18], providing a sufficient theoretical rationale for use of hypochlorous acid in treatment of blepharitis. Many studies have confirmed that hypochlorous acid eye cleansing wipes can effectively reduce the amount of bacteria on the skin around the eyes [19]. Furthermore, studies by our team have shown that hypochlorous acid eyelid wipes have a good effect on blepharitis (including hordeolum); however, the mechanism by which hypochlorous acid acts in the treatment of blepharitis has yet to be clarified. Based on knowledge of the pathogenesis of internal hordeolum and the pharmacological characteristics of hypochlorous acid, we hypothesized that hypochlorous acid eyelid wipes exert their therapeutic function by changing the microbiome of the internal hordeolum, and that understanding the mechanisms involved in hypochlorous acid treatment of internal hordeolum by studying the microbiome could provide new ideas for the application of hypochlorous acid in the field of ophthalmology.

## 2. Methods

### 2.1. Study Population

This study recruited eight patients (2 males and 6 females) with internal hordeolum, who attended the ophthalmology clinic of The First Hospital of Kunming (Southern District) from April 2020 to August 2020. Samples were collected from the subjects before (Group A, 8 samples) and after (Group B, 8 samples) treatment with hypochlorous acid eyelid wipes for 7 days. Microorganisms collected on swabs of secretions from meibomian gland orifices of the two groups were analyzed.

**Figure 1:**
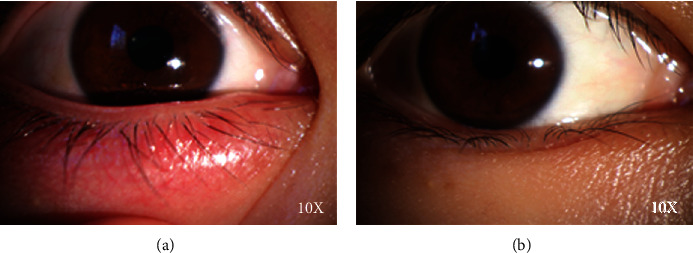
Anterior segment photographs of subjects. (a) Clinical manifestations pretherapy (Group A). (b) Clinical manifestations posttherapy (Group B).

Inclusion criteria were as follows: (1) presented with a painful red lump in one eyelid that appeared spontaneously within 5 days and consented to undergo a complete slit lamp biomicroscope ophthalmic examination; (2) monocular internal hordeolum was diagnosed by the ophthalmologist based on a swollen red eyelid with a painful lump in one eye, bulbar conjunctival hyperemia, telangiectasia, thickening, or irregularity of the eyelid margins, or meibomian gland orifice suppuration [20] (Figure 1(a)). The condition disappeared after 7 days of treatment with hypochlorous acid eyelid wipes (Figure 1(b)); (3) had not received treatment since the onset of the illness, including no use of antibiotics or other drugs and no use of hot or cold compresses or other treatments; and (4) Demodex examination was negative. The exclusion criteria were as follows: (1) lactating or pregnant women, (2) contact lens wearers, (3) combination with other acute ocular inflammation or infection, (4) obvious scarring or keratinization of the eyelid margin, (5) had received eye surgery or eyelid surgery within the past 6 months, (6) use of punctal plugs, (7) Follow-up time exceeded the predetermined time by more than one week, (8) diabetes, glaucoma, and other ocular or systemic diseases that affect the health of the ocular surface, and (9) discrepancies discovered after the start of the experiment (so that the participant met the exclusion criteria). All participants signed the informed consent before participating in the study. This study was approved by The First Hospital of Kunming Institutional Review Board (No. YLS2020-29), and all methods adhered to the principles of the Declaration of Helsinki.

### 2.2. Use of Hypochlorous Acid Eyelid Wipes

All subjects consented to an ophthalmic slit lamp examination when they attended our ophthalmology clinic and then cleaned the eyelid margins with hypochlorous acid eyelid wipes (Xiamen Xinruize, China) once a day for 7 consecutive days. The eyelid margin cleaning method was as follows: wipe the eyelid margin with the convex surface of the wet wipe 10 times, particularly suppuration of the meibomian gland orifices, and then attach the concave surface to the eyelid until the wet wipe dries. Seven days later, the subjects attended the ophthalmology clinic and data were collected from selected cured patients before and after treatment. After topical anesthesia (Santen, Osaka, Japan), pharyngeal swabs were used to collect secretions from the meibomian gland orifices after meibomian gland massage and expression.

### 2.3. Microbiome Analysis

The 16S rDNA gene exists in the genomes of all bacteria and is highly conserved. The sequence contains 9 hypervariable regions and 10 conserved regions. A sequence of a certain hypervariable region (V4 region or V3-V4 region) is amplified by PCR and then sequenced to obtain a sequence of about 1500 bp. The method is as follows: (1) Use DNA extraction kit (MN NucleoSpin 96 Soi) to extract sample DNA; (2) use the following primers (338F 5′-ACTCCTACGGGAGGCAGCA-3′; 806R 5′- GGACTACHVGGGTWTCTAAT-3′) to amplify bacterial 16S V3 + V4 region; (3) microbial diversity library construction and sequencing is as follows: a two-step library construction method, the first step uses DNA as a template, and primers with adapters are designed for PCR, and the second step uses the PCR product of the first step as a template PCR; and (4) microbial diversity analysis: analyze the species composition and diversity change characteristics of the two groups of sample communities through sequencing data quality assessment, OUT analysis, and diversity analysis [21–23].

### 2.4. Statistical Methods

Venn diagrams were used to illustrate the number of common and unique features among samples. The Wilcoxon matched-pairs signed rank test was used to evaluate differences in alpha diversity index between treatment groups. Mothur software and R language tools were used to draw Shannon diversity index dilution curves (the Shannon index reflects the microbial diversity in the sample) for each sample at different sequencing depths. Our experiment included two paired groups of small sample size (where a number of samples ≤20 is defined as a small sample) and the significance of differences between them was analyzed by Wilcoxon rank-sum test. *P* < 0.05 indicated a statistically significant difference. Linear discriminant analysis effect size (LEfSe) analysis was implemented using LEfSe software.

## 3. Results

### 3.1. Taxonomic Assignment

**Figure 2:**
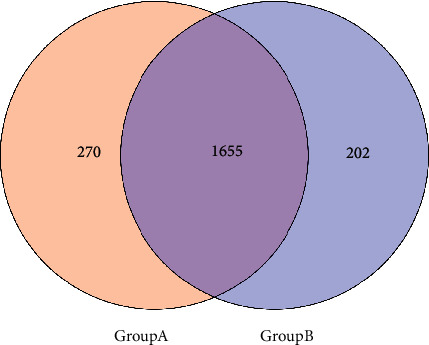
Number of OTUs shared between the two groups.

A total of eight patients with internal hordeolum (2 males and 6 females) were enrolled in this study, and a total of 16 samples were collected before and after eyelid cleaning with hypochlorous acid eyelid wipes, to generate pretherapy (Group A) and posttherapy (Group B) group samples, respectively. Meibomian gland orifice secretion samples were submitted for OTU biodiversity and sample difference analyses before and after treatment (Table 1). The numbers of OTUs in groups A and B were plotted in a Venn diagram, which showed that, of a total of 2127 OTUs, the samples in Group A included 1925 OTUs, and those in Group B 1857 OTUs. (Figure 2).

### 3.2. There is no Significant Change in Bacterial Alpha Diversity of with Internal Hordeolum before and after Treatment with Hypochlorous Acid Eye Cleansing Wipes

**Figure 3:**
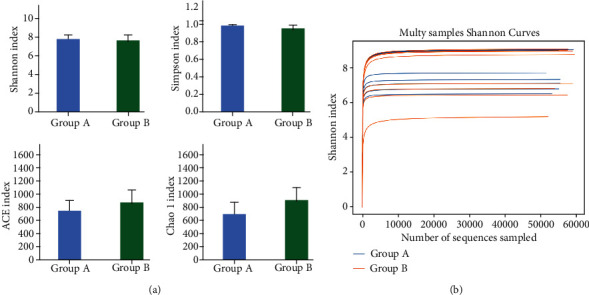
Alpha diversity indices of individual samples. (a) Alpha diversity indices, including Shannon, Simpson, ACE, and Chao1, did not differ significantly between the two groups. (b) All rarefaction curves of individual samples from the internal hordeolum before and after treatment reached the saturation platform, indicating that the sequencing data size was reasonable.

Alpha diversity index analysis was performed on the two groups of samples. Evaluation indicators included the Shannon, Simpson, ACE, and Chao1 indices. Comparisons between the two groups showed no significant difference in any of the indices. This indicates that the microbial diversity was not changed between internal hordeolum onset and cured by the hypochlorous acid eye cleansing wipes. (*P* > 0.05; Table 2; Figure 3).

### 3.3. Microbial Communities from Patients with Internal Hordeolum before and after Treatment

**Figure 4:**
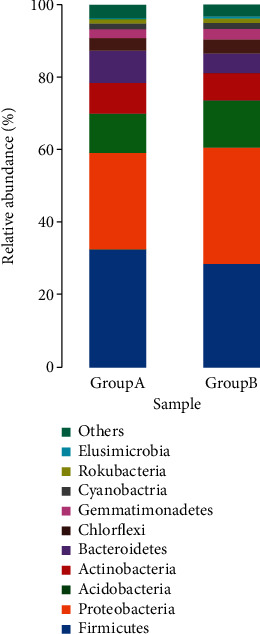
The relative abundances of core phyla before and after treatment. (A) The relative abundances of the top 10 most abundant phyla. There was no significance between the two groups at the phylum level (*P* > 0.05).

We detected the relative abundance of OTUs in microbial communities from the patients with internal hordeolum before and after treatment with a hypochlorous acid eyelid wipe. The major phyla detected before treatment included *Firmicutes* (32.78% ± 20.16%), *Proteobacteria* (26.73% ± 7.49%), *Acidobacteria* (10.58% ± 11.45%), *Bacteroidetes* (9.05% ± 6.63%), *Actinobacteria* (8.48% ± 1.77%), and *Chloroflexi* (3.15% ± 3.12), and those after treatment were as follows: *Proteobacteria* (31.86% ± 9.69%), *Firmicutes* (29.07% ± 24.20%), *Acidobacteria* (11.33% ± 7.53%), *Actinobacteria* (7.10% ± 1.98%), *Bacteroidetes* (5.39% ± 5.17%), and *Chloroflexi* (3.89% ± 3.67%). There was no significant difference between the two groups at this level (Figure 4).

Significant differences in bacterial communities were detected between the two groups at the class level. At the genus level, compared with Group B, Group A had a significantly higher abundance of *Staphylococcus* (1.58% ± 3.5% vs. 0.17% ± 0.19%; *P* < 0.05), *Ruminococcaceae_UCG014* (0.88% ± 0.82% vs. 0.21% ± 0.37%; *P* < 0.05), *Neisseria* (0.37% ± 0.45% vs. 0.13% ± 0.36%; *P* < 0.05), *Methylobacterium* (0.35% ± 0.32% vs. 0.07% ± 0.05%; *P* < 0.05), *Massilia* (0.29% ± 0.20% vs. 0.18% ± 0.23%; *P* < 0.05), with lower abundances of *Pantoea* (0.05% ± 0.12% vs. 0.14% ± 0.17%; *P* < 0.05), *Subdoligranulum* (0.03% ± 0.04% vs. 0.50% ± 0.85%; *P* < 0.05), and *Ruminococcaceae_UCG-002* (0.02% ± 0.04% vs. 0.04% ± 0.09%; *P* < 0.05) (Figure 4). This shows that the differences of the microorganisms between the onset and the cure to internal hordeolum start from the class level.

### 3.4. Pathogens such as Staphylococcus and Neisseria are Reduced after Treatment; Faecalibacterium prausnitzii and other Probiotics are Increased Compared to Pretherapy Group

LEfSe software was used for multilevel species discrimination of high-dimensional biomarkers for internal hordeolum and analysis of genomic characteristics before and after treatment, and significant differences were detected (*P* < 0.05). Linear discriminant analysis (effect size) indicated that the core flora in the Group A microbial community were *c-Actinobacteria*, *f-Staphylococcaceae*, *g-Staphylococcus*, *s-S. aureus*, *g-Ruminococcacea-UCG-014*, *s-Ruminococcacea-UCG-014*, *f-Halomonadaceae*, *g-Neisseria*, *f-Methylobacterium*, *o-Frankiales*, and *s-Neisseria sicca*, whereas those in Group B were *s-Streptococcus* sp., *g-Blautia*, *s-Bifidobacterium pseudocatenulatum*, *g-Subdoligranulum*, *s-Subdoligranulum variabile*, *g-Faecalibacterium*, and *s-Faecalibacterium prausnitzii*. It is speculated that hypochlorous acid eye cleansing wipes in the treatment of hordeolum can be effective by reducing the relative abundance of pathogenic bacteria and increasing the relative abundance of probiotics. (prefix explanation: *c-* means class level*; o-* means order level; *f-* means family level; *g-* means genus level; and *s-* means specie level.)

## 4. Discussion

The significance of the ocular microbiome in eye health has become a research focus in ophthalmology in recent years, primarily concerned with the ocular surface microbiome (OSM) [24]. Many studies have reported significant differences in the microbiomes of patients with ocular surface diseases such as blepharitis, (including internal hordeolum), meibomian gland dysfunction, dry eye, and keratitis, compared with healthy people [25–29]; however, there has been a lack of comparative studies of microorganisms in these diseases before and after treatment, and investigating the changes in microbial communities will be helpful in understanding the pathogenesis of internal hordeolum, as well as prevention and treatment methods.

The biodiversity and relative abundance of organisms in the ocular surface microbiome are affected by age, sex, geographic differences, and microbial habitat [30–32]. The mean age of the subjects in this study was 28.38 ± 4.24 years old. A paired sample test method was used to collect secretions from the meibomian glands before and after treatment for microbial high-throughput sequencing, which avoided the influence of other factors on the study results. In groups A and B, 1955 and 1857 OTUs were identified, respectively. There was no significant difference in OTUs before and after treatment for internal hordeolum with hypochlorous acid eyelid wipes. The Shannon index was used to draw a dilution curve to analyze the complexity of the two sets of samples, and the results, that its, platform curve, showed that the coverage depth was >99%, indicating that the sequencing data reflected microorganism diversity information relatively completely.

We found no significant differences in alpha diversity between the two groups, according to the analysis of the Shannon, Simpson, ACE, and Chao1 indices (*P* > 0.05), indicating that the microbial communities in the two groups were highly similar and that the composition of the microbiome did not alter before and after treatment. Moreover, we detected no significant difference in flora before and after treatment at the phylum level. The major phyla in patients with internal hordeolum before treatment included *Firmicutes* (32.78% ± 20.16%), *Proteobacteria* (26.73% ± 7.49%), *Acidobacteria* (10.58% ± 11.45%), *Bacteroidetes* (9.05% ± 6.63%), *Actinobacteria* (8.48% ± 1.77%), and *Chloroflexi* (3.15% ± 3.12%), while after treatment the major phyla were as follows: *Proteobacteria* (31.86% ± 9.69%), *Firmicutes* (29.07% ± 204.20%), *Acidobacteria* (11.33% ± 7.53%), *Actinobacteria* (7.10% ± 1.98%), *Bacteroidete*s (5.39% ± 5.17%), and *Chloroflexi* (3.89% ± 3.67%). Dong et al. demonstrated that, among healthy people, the core flora are *Proteobacteria* (64%), *Actinomycetes* (19.6%), and *Firmicutes* (3.9%) [33], while Delbeke conducted a review of all current research data after calculation and integration and found that 16S rRNA detection from ocular surface core bacteria in healthy people included mainly *Actinomycetes* (53%), followed by Proteobacteria (39%) and *Firmicutes* (8%) [34]. The core flora types detected at the phylum level in this study are consistent with the findings described above; however, the relative abundance ratio differed, indicating that changes in the relative abundance of organisms in the OSM are part of internal hordeolum pathogenesis, and restoration of OSM to a healthy state is key to treatment. Combined with our results showing that treatment with hypochlorous acid eyelid wipes did not change the bacterial diversity of meibomian gland secretions before and after treatment, and the differences in the dominant flora before and after treatment, our data support this hypothesis.

There were statistically significant differences in the relative abundances of bacterial communities between groups A and B beginning at the class level. LEfSe analysis was used to identify dominant species with significant differences in abundance between the two groups. The dominant flora in Group A were *c-Actinobacteria*, *f-Staphylococcaceae*, *g-Staphylococcus*, *s-S. aureus*, *g-Ruminococcacea-UCG-014*, *s-Ruminococcacea-UCG-014*, *f-Halomonadaceae*, *g-Neisseria*, *f-Methylobacterium*, *o-Frankiales*, and *s-Neisseria sicca.* Previous studies have proven that the purulent reaction in the internal hordeolum is caused by *S. epidermidis* or *S. aureus,* invasion of the meibomian glands [3], which is consistent with our results; however, in addition to *Staphylococcus*, we also found that the abundance levels of *Neisseria*, *Actinomycetes*, and *Ruminococcus* were also decreased after treatment, which has not been reported in previous studies. Overall, our results suggest the following two points: (1) Other microorganisms may be involved in the occurrence of internal hordeolum. Hypochlorous acid eyelid wipes have a therapeutic effect by reducing the relative abundance of the above-mentioned symbiotic pathogens. At present, the drugs most routinely used in ophthalmology are quinolone antibiotics; however, the first choice for the treatment of *Neisseria* is *β*-lactam antibiotics such as penicillin [35]. Our results likely explain the frequent occurrence of drug resistance in the clinic. Hypochlorous acid can inactivate microorganisms by oxidizing ATP hydrolases and synthetases to prevent the production of energy-providing ATP [36]. The results of this study also support that this ingredient can effectively kill a variety of pathogenic bacteria that cause internal hordeolum. (2) The reason for our detection of novel commensal pathogenic bacteria may be related to advances in the detection method. Previous studies of ophthalmic microorganisms have been limited to the use of the culture detection method, which can only detect bacteria grown in standardized laboratories. It is estimated that most bacteria cannot be cultured in the laboratory; however, high-throughput sequencing methods can detect more of the microbiome. High-throughput sequencing studies have found that the core microbiome of the ocular surface is very different from that detected using traditional culture methods and contains numerous bacteria that had never been described previously [30, 34, 37], consistent with the results of this study.

The dominant flora in Group B were *s-Streptococcus* sp., *g-Blautia*, *s-Bifidobacterium pseudocatenulatum*, *g-Subdoligranulum*, *s-Subdoligranulum variabile*, *g-Faecalibacterium*, and *s-F. prausnitzii*. In other studies, no prominent indications of these types of probiotic organisms have been reported in meibomian gland secretions from healthy people, indicating that the meibomian glands analyzed in the treated state may contain different microbes from those found in healthy people. It is worth paying attention that the relative abundance of *F. prausnitzii* increased after the disease is cured in our study for the first time. Since there is no healthy control group in this paper, and there are no researches reported on hypochlorous acid regulating the relative abundance of *F. prausnitzii*, it is indeed impossible to determine whether the changes of bacteria are caused by the self-healing process of the human body or induced by hypochlorous acid. However, we speculate that there is a possibility that hypochlorous acid regulates the relative abundance of *F. prausnitzii* increased to treat the disease. The reasons are as follows: (1) *F. prausnitzii* is a major component of the intestinal flora and has been confirmed by numerous studies as a biological indicator of human health. *F. prausnitzii* produces energy and anti-inflammatory metabolites that can support host health. This bacterium is among the most important butyrate-producing microbes in the human colon [38]. Its metabolites, butyrate, and salicylic acid block NFkB signal transduction, which is a regulator of inflammation [39]. Decreases in the *F. prausnitzii* population promote inflammatory processes [40], and some studies have shown that the relative abundance of *F. prausnitzii* in dry eye and keratitis is lower than healthy people [41]. There are studies showed that the relative abundance of F. prausnitzii has increased after treatment with tacrolimus and other therapeutic drugs, which suggest that the *F. prausnitzii* are changed after the treatment of the disease [42, 43]. This indicates that the relative abundance of *F. prausnitzii* may be induced to increase in the process of drug treatment to play an anti-inflammatory role.(2) In addition to the broad-spectrum sterilization effect, hypochlorous acid can also reduce the levels of inflammatory factors [11]. There may be some potential connection between these changes. In summary, our results suggest that increasing the relative abundance of probiotics, such as *F. prausnitzii*, is potentially related to the anti-inflammatory effects of hypochlorous acid.

In conclusion, the core microbiome in the internal hordeolum includes *Firmicutes*, *Proteobacteria*, *Acidobacteria*, *Bacteroidetes*, *Actinobacteria*, and *Chloroflexi* consistent with the organisms found in the OSM, albeit at different relative abundances. Hypochlorous acid eyelid wipes do not change the microbial diversity of meibomian gland secretions before and after eyelid margin cleaning. The potential mechanism underlying the effects of hypochlorous acid eyelid wipes could be reduction of the relative abundance of commensal pathogenic bacteria, such as *Staphylococcus, Neisseria, Actinomycetes,* and *Ruminococcus* in patients with internal hordeolum via its broad-spectrum antibacterial effects, leading to increases in the relative abundance of *F. prausnitzii* and other probiotics, to mediate anti-inflammatory therapy for internal hordeolum. Considering the small sample size of this study and in order to ensure the accuracy of the research, subsequent studies with increased sample size and adding control group were necessary to make a solid conclusion about the mechanism of hypochlorous acid eyelid wipes on internal hordeolum.

## Figures and Tables

**Table 1:** Summary sequencing data from the two paired group.

Group	Subject	Subject no.	Age	Sex	Reads	High quality reads	Average read length (bp)	OTUs
Pretherapy group (group A)	StOD0d2	1	32	Female	80180	65539	417	316
	StOD0d3	2	24	Female	80068	62882	417	319
	StOS0d4	3	28	Female	79539	64427	418	311
	StOD0d5	4	29	Male	79610	65553	420	233
	StOD0d11	5	29	Female	79995	76296	418	1193
	StOD0d12	6	26	Female	79687	75793	418	1231
	StOD0d13	7	23	Male	80347	76903	418	1197
	StOD7d3	8	36	Female	80185	63218	419	191

Posttherapy group (group B)	StOD7d4	1	32	Female	79660	65596	417	220
	StOD7d2	2	24	Female	79922	67809	417	268
	StOS7d1	3	28	Female	79731	65019	421	203
	StOD7d8	4	29	Male	79845	76476	418	1194
	StOD7d6	5	29	Female	80154	76856	418	1200
	StOD7d7	6	26	Female	80396	73965	421	1302
	StOD7d5	7	23	Male	79834	76891	418	1234
	StOD2m1	8	36	Female	80875	76929	418	1184

^∗^St indicates staphylococcal blepharitis, which is equivalent to internal hordeolum in this study. OD, right eye; OS, left eye; 0d/7d3, Pretherapy group (Group A); 7d/2 m1, Posttherapy group (Group B); no., serial number.

**Table 2:** Comparison of differences in *α* diversity index between the two groups.

Alpha diversity	Group A	Group B	*P* value
Shannon	7.521[6.879,9.036]	7.935[6.540,8.987]	*P* > 0.05
Simpson	0.992[0.983,0.996]	0.988[0.980,0.996]	*P* > 0.05
ACE	656.6[344.4,1238]	1214[244.2,1259]	*P* > 0.05
Chao1	408.2[307.3,1290]	1257[263.1,1302]	*P* > 0.05

## Data Availability

The 16S rRNA high-throughput sequencing of meibomian gland secretions from patients with internal hordeolum used to support the findings of this study is available from the corresponding author upon request.
